# Phase Transformation and Mechanical Optimization of Eggshell-Derived Hydroxyapatite across a Wide Sintering Temperature Range

**DOI:** 10.3390/ma17164062

**Published:** 2024-08-15

**Authors:** Shih-Ching Wu, Hsueh-Chuan Hsu, Mei-Yi Liu, Wen-Fu Ho

**Affiliations:** 1 Department of Dental Technology and Materials Science, Central Taiwan University of Science and Technology, Taichung 406053, Taiwan; scwu@ctust.edu.tw (S.-C.W.); hchsu@ctust.edu.tw (H.-C.H.); 2 Department of Materials Science and Engineering, Da-Yeh University, Changhua 515006, Taiwan; 3 Department of Chemical and Materials Engineering, National University of Kaohsiung, Kaohsiung 811726, Taiwan

**Keywords:** antibacterial activity, eggshell, hydroxyapatite, mechanical properties, sintering temperature

## Abstract

Calcium phosphate, particularly hydroxyapatite (HA), bears a close resemblance to human bones, rendering it a prevalent material in biomedical applications. This study focuses on the successful preparation of HA using a precipitation method with eggshell as a raw material. Subsequently, the HA powder was press-formed and sintered at various temperatures to investigate the impact of sintering temperature on the mechanical properties, including hardness, compressive strength, and fracture toughness, of the sintered HA samples (E-HA). Statistical analyses, including one-way ANOVA and Tukey’s post-hoc test, were conducted to determine significant differences in these properties at different sintering temperatures. Experimental findings revealed that as the sintering temperature increased, HA partially transformed into β-TCP between 800 and 1300 °C, with α-TCP observed at 1400 °C. The elimination of pores led to an increase in relative density, with a maximum relative density of 94.5% achieved at 1200 and 1300 °C. E-HA sintered at 1200 °C exhibited the highest hardness (5.08 GPa), compressive strength (255.79 MPa), and fracture toughness (1.21 MPa·m0.5). However, at 1400 °C, a slight decrease in apparent density (2.90 g/cm^3^) was noted due to the presence of α-TCP, along with significant grain growth. This study’s objective is clearly aligned with the study design, incorporating detailed statistical analyses to validate the findings. Furthermore, bacterial culture experiments were conducted using sintered E-HA, Chem-HA (HA synthesized from reagent-grade calcium carbonate), and Comm-HA (commercial HA). *Streptococcus mutans* was cultured on the surfaces of sintered E-HA, Chem-HA, and Comm-HA samples for 20 h. After culturing, the OD values for all samples were below 0.2, indicating significant antibacterial efficacy. The comparable OD values and bacterial counts (*p* > 0.05) suggest that the source of HA does not impact its antibacterial properties. This underscores the potential of eggshell-derived HA as an effective material for biomedical applications.

## 1. Introduction

With the rapid advancement of nanotechnology, various nanomaterials, such as calcium phosphate ceramics and silicon-based nanomaterials, have been applied in bone repair and regeneration. Silicon-based nanomaterials, particularly self-assembled silica nanowebs, exhibit both drug delivery and extracellular matrix characteristics, enhancing cellular attraction and proliferation [1]. Calcium phosphate ceramics, including hydroxyapatite (HA), β-tricalcium phosphate (β-TCP), α-tricalcium phosphate (α-TCP), tetracalcium phosphate (TTCP), and amorphous calcium phosphate (ACP), are chemically stable and structurally similar to bone minerals. Among these, HA (Ca_10_(PO_4_)_6_(OH)_2_), with its ideal calcium-to-phosphorus molar ratio of 1.67, is the most representative calcium phosphate ceramic, exhibiting excellent bioactivity, biocompatibility, and osteoconductivity [2,3]. This enables HA to form strong bonds with natural bone, making it highly suitable for dental and orthopedic uses. Natural bone HA contains small amounts of carbonate (approximately 3–8%) [4] and trace elements such as Na, Mg, and Sr [5].

The preparation of bulk HA materials for load-bearing applications involves forming and sintering HA powders. Increasing sintering temperature or time enhances the density of HA compacts, thereby improving their compressive strength, hardness, and fracture toughness. However, excessive grain growth during sintering can inversely affect these mechanical properties [6,7]. Thus, optimizing sintering conditions is crucial for maximizing the mechanical properties of HA. Techniques like liquid-phase sintering with low-melting-point second phases can enhance HA properties, although these are typically used for HA composites with materials like phosphate glass or silicate glass [8,9,10]. Recently, microwave sintering has shown promise in improving HA mechanical properties by achieving dense HA blocks in a shorter time, although its high cost and low production yield limit its widespread use [11].

In recent years, there has been significant research interest in nano-sized HA. Nano-HA has emerged as a versatile material due to its excellent biocompatibility and structural similarity to inorganic bone. HA nanoparticles possess a large surface area that enhances osteoclast-like cell functions and closely resembles the structure of bone HA [12]. Smaller HA powder grains lead to smaller grain sizes in sintered compacts, resulting in higher compressive strength, microhardness, and fracture toughness [13]. Studies by Kalita et al. [14] and Ramesh et al. [6] have demonstrated that nano-sized HA achieves greater density and lower sintering temperatures compared to micron-sized HA due to enhanced heat conduction and diffusion facilitated by the large surface area of nano-crystalline HA.

In previous studies, our research team successfully synthesized HA from natural calcium-containing waste materials such as eggshells and oyster shells. We employed various methods including hydrothermal processes [15], microwave irradiation [16], and aqueous precipitation [17]. Notably, HA derived from eggshells demonstrated remarkable bone-induction properties in animal experiments. The results showed extensive new bone growth with abundant Haversian canals, and nearly all HA granules were enveloped by newly formed bone tissue, indicating an excellent osteoinductive capability [18]. Additionally, HA synthesized from oyster shells was utilized as a bioactive coating on titanium metal surfaces [19], further demonstrating its versatile applications in biomedical fields. Additionally, we explored the use of natural plant extracts as templates to control HA grain size and morphology [20]. The resulting HA powders from these calcium-containing natural waste materials exhibited nano-scale structures with carbonates and trace elements (Mg and Sr), closely resembling human bone composition.

This study specifically focuses on synthesizing HA powder from eggshells using the aqueous precipitation method, followed by press-forming and sintering at various temperatures. While the use of eggshells for HA synthesis is established, the research introduces novelty by systematically investigating the impact of a broad range of sintering temperatures (800–1400 °C) on the mechanical properties of HA compacts. This comprehensive temperature study provides new insights into how temperature variations influence microhardness, compressive strength, and fracture toughness, which is less-commonly explored in the existing literature. Additionally, our study uniquely combines mechanical and antibacterial assessments, evaluating both the strength and antibacterial efficacy of HA. Addressing the null hypothesis that there is no significant difference in the mechanical properties of HA scaffolds sintered at different temperatures provides targeted insights into the effects of sintering conditions. This approach enhances the understanding of HA material optimization and its potential applications in biomedical fields, highlighting the study’s relevance for the development of advanced biomaterials.

## 2. Materials and Methods

### 2.1. Synthesis of HA by Precipitation Method

To begin, 2.0 g of eggshell powder was placed in a beaker, followed by the addition of an aqueous acetic acid solution (25 vol%) to dissolve the eggshell. The mixture was stirred with a magnetic stirrer until the eggshell was completely dissolved. Then, (NH_4_)_2_HPO_4_ (Showa, Osaka, Japan) solution (24.06 wt%) was added. The pH of the solution was adjusted to 10 using NH_4_OH (Showa, Osaka, Japan), and the precipitation reaction was allowed to proceed for 24 h. The resulting HA suspension was poured into a larger beaker and rinsed repeatedly with deionized water several times. After suction filtration using a Whatman filter paper (pore size 2.5 µm) (Cytiva, Marlborough, MA, USA), the precipitates were placed in an oven at 45 °C for 12 h. The dried precipitates were then ground into a fine powder using an agate mortar and pestle. In this experiment, E-HA refers to HA prepared by the above process.

### 2.2. Sintering of HA

The HA powder synthesized from eggshells (E-HA) was first press-molded into green compacts using a uniaxial hydraulic press. Specifically, E-HA powder was placed into a stainless-steel die and subjected to a pressure of 100 MPa for 5 min to form cylindrical green compacts. These green compacts were then sintered in a high-temperature furnace at various temperatures (800 °C, 900 °C, 1000 °C, 1100 °C, 1200 °C, 1300 °C, and 1400 °C) for 4 h each to investigate the impact of sintering temperature on the microstructure and mechanical properties. The dimensions of the samples used for XRD, SEM, microhardness, fracture toughness, and density measurements were 13.0 mm in diameter and 2.5 mm in thickness. These dimensions were chosen to ensure consistency in measurement and to provide an adequate sample size for analysis. The samples for the compressive strength test had a diameter of 7.0 mm and a height of 12.7 mm. This specific size was selected to ensure that the length-to-diameter ratio fell within the range between 1.5 and 2.0. This control of the length-to-diameter ratio is crucial for minimizing edge effects and ensuring uniform stress distribution during the test [21]. For the antibacterial test, the samples were 13.0 mm in diameter and 1.0 mm in thickness. This thickness is commonly used in antibacterial assays to ensure proper contact with the bacteria and accurate assessment of antibacterial properties.

### 2.3. X-ray Diffraction Analysis, Phase Content, and Crystallinity

The compact samples of E-HA, sintered at various temperatures, were subjected to phase analysis using an X-ray diffractometer (XRD, XRD-6000, Shimadzu, Kyoto, Japan). The analysis employed Ni-filtered Cu Kα radiation (λ = 1.5406 Å) with an operating voltage of 30 kV and an operating current of 30 mA. The scanning region was set from 2θ = 20° to 50° with a scanning speed of 2°/min and a step size of 0.02°. The phase contents of samples sintered at different temperatures were calculated from the integrated peak areas according to the main diffraction peaks of HA, β-TCP, and α-TCP, corresponding to the (211), (0210), and (170) planes, respectively [22,23]. The crystallinity (X_c_) of HA was calculated using the following formula [24]:

(1) $$X_{c}=1-\frac{V_{112/300}}{I_{300}}$$

where I_300_ is the intensity of the (300) plane, and V_112/300_ is the intensity of the hollow between the (112) and (300) planes.

### 2.4. Microstructural Observations and Grain Size Calculation

The microstructures of the E-HA samples sintered at various temperatures were observed using a field-emission scanning electron microscope (FE-SEM, JSM-7401F, JEOL Ltd., Tokyo, Japan). The E-HA samples were sequentially ground with 400-, 800-, 1200-, 1500-, 2000-, and 4000-grit silicon carbide abrasive papers, followed by polishing with diamond lapping film using a diamond grain size of 1 μm. Subsequently, hot etching was performed for 15 min at temperatures 100 °C lower than the respective sintering temperatures. The grain size was measured according to the Heyn linear intercept procedure as outlined in the ASTM E112-96 standard [25].

### 2.5. Compressive Strength Measurements

The compressive strengths of the E-HA samples sintered at various temperatures were tested in accordance with ASTM C1424-10 [26]. Each sample was placed on a universal testing machine (AG-IS, Shimadzu, Japan) and subjected to a cross-head speed of 1 mm/min until fracture. At least five specimens were tested for each sintering condition, and the average value was calculated.

### 2.6. Apparent Density Measurements

The apparent densities of the E-HA specimens sintered at various temperatures were measured using the Archimedes method with distilled water as the immersion medium. The relative densities of the E-HA samples were calculated based on the theoretical density of HA (3.16 g/cm^3^) [27]. The apparent density (ρ_a_) of the sample was calculated using the following formula [28]:

(2) $$\rho_{a}=\frac{W_{1}}{(W_{3}-W_{2})/\rho_{\mathrm{water}}}$$

where ρ_a_ (g/cm^3^) is the apparent density of the sample, W_1_ (g) is the dry weight, W_2_ (g) is the suspended weight measured in deionized water, W_3_ (g) is the saturated weight, and ρ_water_ is the density of water (1 g/cm^3^).

### 2.7. Microhardness Testing

Disc-shaped samples of the E-HA (13.0 mm in diameter and 2.5 mm in thickness), sintered at various temperatures, were sequentially ground with 400-, 800-, 1200-, 1500-, 2000-, and 4000-grit silicon carbide abrasive papers. They were then polished using diamond lapping film with a diamond grain size of 1 μm. The Vickers microhardness test was conducted using a microhardness tester (HMV-2, Shimadzu, Tokyo, Japan) under a load of 200 g for 10 s. At least five different positions were measured for each specimen, and at least five specimens were tested for each sintering condition. The average hardness value was then calculated.

### 2.8. Fracture Toughness Evaluation

The fracture toughness of the E-HA samples sintered at various temperatures was evaluated by measuring the crack length of the indentation left on the sample surface after the hardness test. The fracture toughness (K_Ic_) was calculated using the following formula [29]:

(3) $$K_{Ic}=0.203{\left(\frac{c}{a}\right)}^{-1.5}HV{\left(a\right)}^{0.5}$$

where c is the average crack length (μm) of the indentation, a is the average half-diagonal length of the indentation (μm), and HV is the microhardness value (GPa).

### 2.9. Antimicrobial Activity Assessment

This study investigated the antibacterial activity of the E-HA samples sintered at 1200 °C. Additionally, samples synthesized from chemical reagent-grade calcium carbonate (Chem-HA) and commercially available HA samples (Comm-HA) were used as control groups, also sintered at 1200 °C. E-HA, Chem-HA, and Comm-HA samples were each co-cultured with *Streptococcus mutans* to evaluate their antibacterial activity.

First, *Streptococcus mutans* was cultured on agar plates for 12 h at 37 °C. Single colonies were then inoculated into Todd–Hewitt broth (THB) and incubated in a shaker incubator for 20 h at 37 °C. Subsequently, 150 μL of bacterial culture was further incubated in the shaker incubator for 6 h. The bacterial cells were then separated from the culture medium by centrifugation at 8000 rpm for 10 min. The pellet was resuspended in fresh THB, and bacterial concentration was measured spectrophotometrically using a spectrophotometer. Bacterial suspensions with a concentration of 0.5 OD were co-cultured with the test samples for 20 h. After co-cultivation, the samples were rinsed three times with sterile water, stained with crystal violet solution for 10 min, and then rinsed again three times with sterile water. Decolorization was performed using 95% ethanol for 15 min. After decolorization, ethanol was transferred to a 96-well culture plate, and the absorbance was read at a wavelength of 595 nm using an enzyme-linked immunosorbent assay (ELISA) reader (Multiskan GO, Thermo Scientific, Waltham, MA, USA).

### 2.10. Experimental Design and Statistical Analysis

To determine the sample size for our experimental tests, we followed standard practices and guidelines in materials science research. For mechanical property evaluations, at least five specimens were tested per condition to ensure reliable statistical analysis. For antimicrobial activity assessments, three specimens were used for each condition. This approach accounts for variability and ensures the accuracy and validity of our findings. The summary of experimental groups, sample numbers, and tests performed is shown in Table 1.

The compressive strength, microhardness, fracture toughness, relative density, and grain size data were analyzed using one-way ANOVA followed by Tukey’s post-hoc test to identify significant differences among HA samples sintered at varying temperatures. Antimicrobial activity was assessed using one-way ANOVA to compare bacterial counts among E-HA, Chem-HA, and Comm-HA samples. Statistical significance was set at *p* < 0.05 or 0.01. In the figures, * indicates *p* < 0.05, and ** indicates *p* < 0.01.

## 3. Results

### 3.1. XRD Analysis of HA Post-Sintering

**Figure 1:**
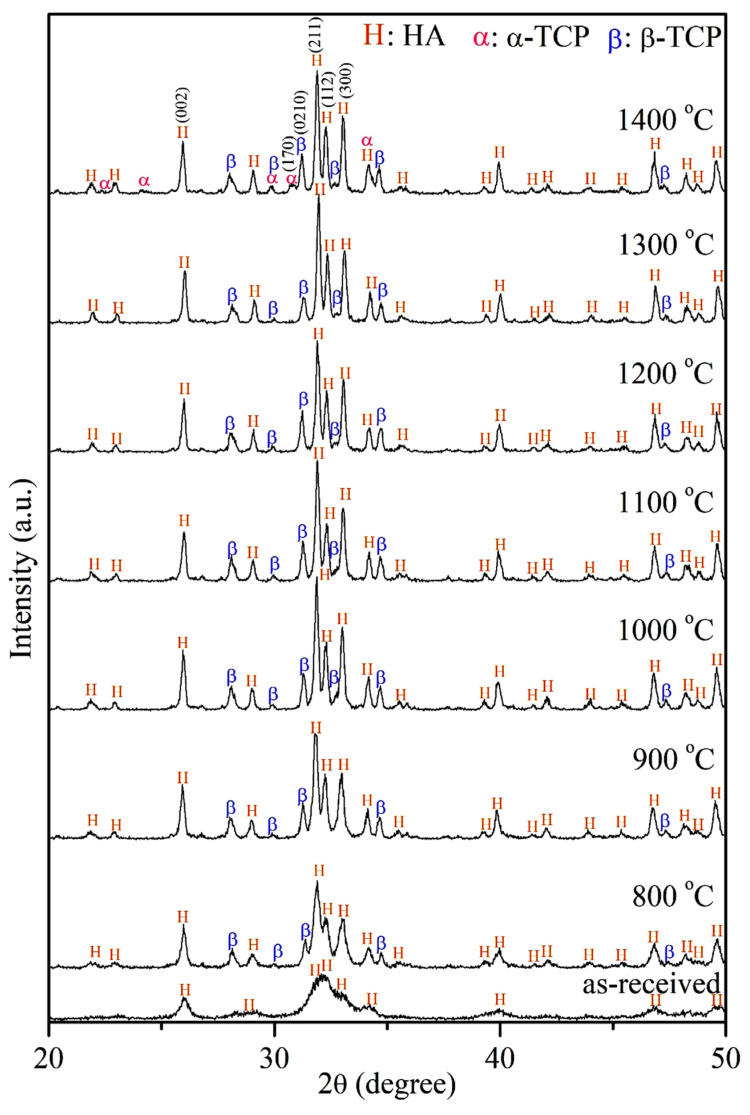
XRD patterns of E-HA green compacts (as-received) and their blocks, sintered at 800–1400 °C.

The X-ray diffraction (XRD) patterns of E-HA green compacts and their blocks sintered at temperatures ranging from 800 to 1400 °C are shown in Figure 1. It was observed that unsintered E-HA green compact consisted of a single-phase HA without any other crystal phases. Due to its low crystallinity and nanoscale size, the diffraction peaks appeared broadened. Upon sintering E-HA at temperatures between 800 and 1400 °C, a phase transformation to β-TCP was evident. At 1400 °C, a small amount of α-TCP phase was also observed, indicating further transformation at higher temperatures. The proportion of β-TCP phase ranged from 19% to 27% across the various sintering temperatures, while that of the α-TCP phase was less than 10% in samples sintered at 1400 °C. The initial crystallinity of the E-HA green compact was measured at 32%. As the sintering temperature increased, the diffraction peaks sharpened, indicating an increase in crystallinity. Specifically, after sintering at 800 °C and 900 °C, the crystallinity of E-HA samples rose to 78% and 89%, respectively. Sintering temperatures exceeding 1000 °C resulted in a crystallinity of approximately 95%.

### 3.2. Relative Density

**Figure 2:**
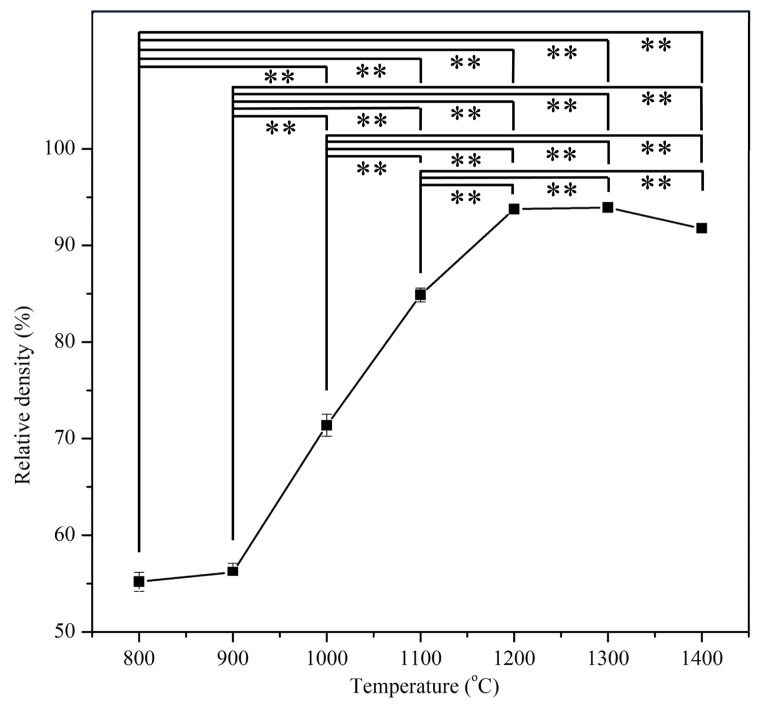
Relative densities of E-HA blocks sintered at different temperatures. ** indicates *p* < 0.01.

The apparent densities of the samples after sintering in this study ranged from approximately 1.74 to 2.96 g/cm^3^, significantly lower than the theoretical density of HA (3.16 g/cm^3^). The relative density was calculated using the apparent density of E-HA compacts sintered at various temperatures and the theoretical density of HA, as depicted in Figure 2. As the sintering temperature increased (from 800 to 1200 °C), the relative density of the compacts increased from 55.5% (at 800 °C) to 94.5% (at 1200 °C), reflecting the consolidation process. Temperatures above 1200 °C resulted in compacts with relative densities exceeding 90%, indicating effective densification. However, at 1400 °C, a slight decrease in relative density was observed. This temperature range coincided with the observation of α-TCP, which has a lower density (2.86 g/cm^3^) compared to HA (3.16 g/cm^3^), contributing to the minor reduction in relative density of the sintered compact.

### 3.3. Microstructural Observation

**Figure 3:**
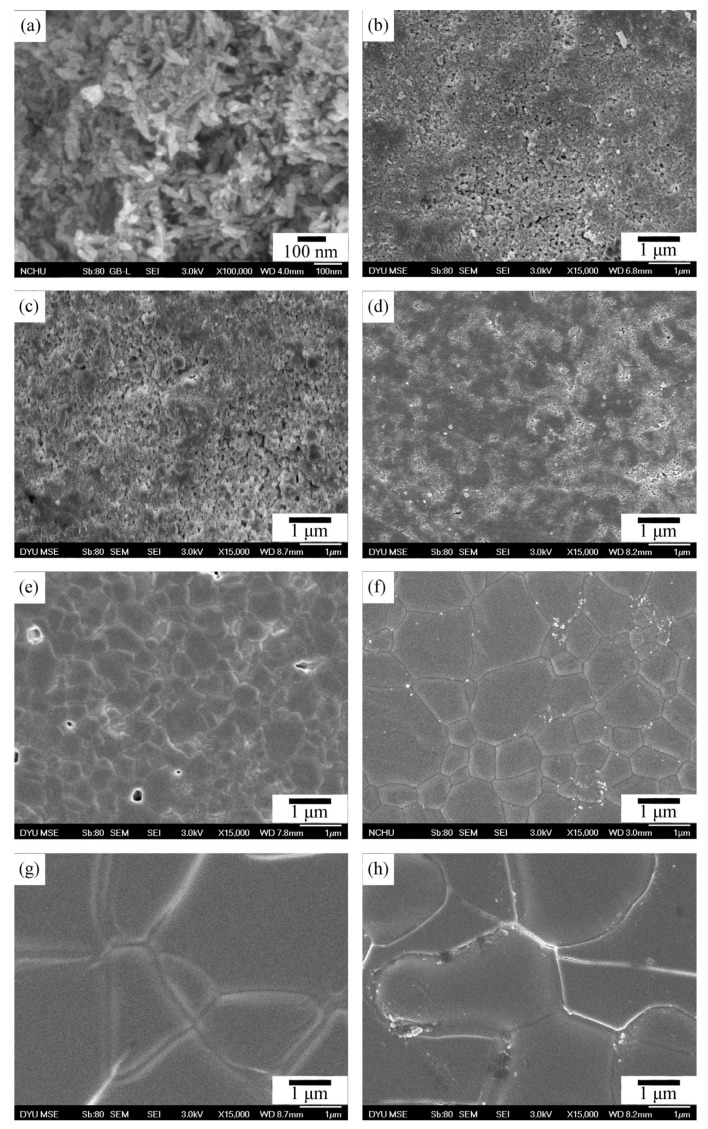
SEM images of E-HA powders (**a**) and their compacts after sintering at 800 °C (**b**), 900 °C (**c**), 1000 °C (**d**), 1100 °C (**e**), 1200 °C (**f**), 1300 °C (**g**), and 1400 °C (**h**).

Figure 3 presents FE-SEM photographs of E-HA powders and compacts after sintering at temperatures ranging from 800 to 1400 °C. In this study, E-HA synthesized via the precipitation method exhibited nano-sized rod-shaped particles, as depicted in Figure 3a, with an average particle size of 82.67 ± 5.58 nm in length and 33.33 ± 0.01 nm in width. Upon sintering at 800 °C and 900 °C (Figure 3b,c), necking between particles began to form, indicating that initial stages of densification were not yet complete. At 1000 °C (Figure 3d), existing pores in the sample were largely eliminated, marking the onset of significant densification, correlating with the observed increase in relative density (Figure 2). Although some small pores remained visible, clear grain boundaries were evident in samples sintered at 1100 °C (Figure 3e), contributing to an increase in E-HA relative density to 85.5%. Sintering at 1200 °C (Figure 3f) resulted in the nearly complete elimination of pores, achieving the highest relative density (94%). However, at higher sintering temperatures of 1300 °C and 1400 °C (Figure 3g,h), significant grain growth was observed.

**Figure 4:**
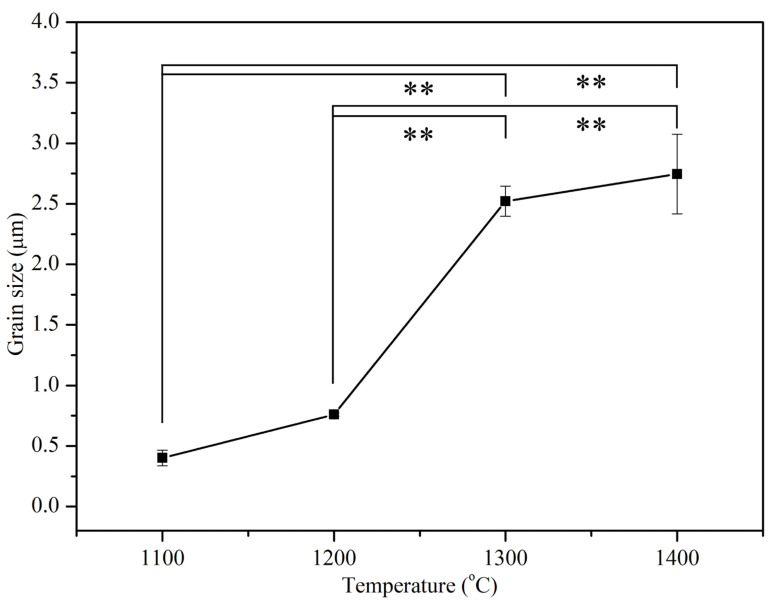
Grain sizes of E-HA blocks sintered at different temperatures. ** indicates *p* < 0.01.

Figure 4 illustrates the grain sizes obtained from SEM photographs of E-HA compacts sintered from 1100 to 1400 °C. At lower sintering temperatures (800 to 1000 °C), grain boundaries were not clearly observed due to insufficient densification. Above 1100 °C, distinct grain boundaries began to appear, with grain size increasing as sintering temperature increased. At 1400 °C, the largest average grain size observed was approximately 2.746 μm. Sintering temperatures exceeding 1200 °C exhibited substantial grain coarsening, leading to significantly larger grain sizes.

### 3.4. Mechanical Properties

**Figure 5:**
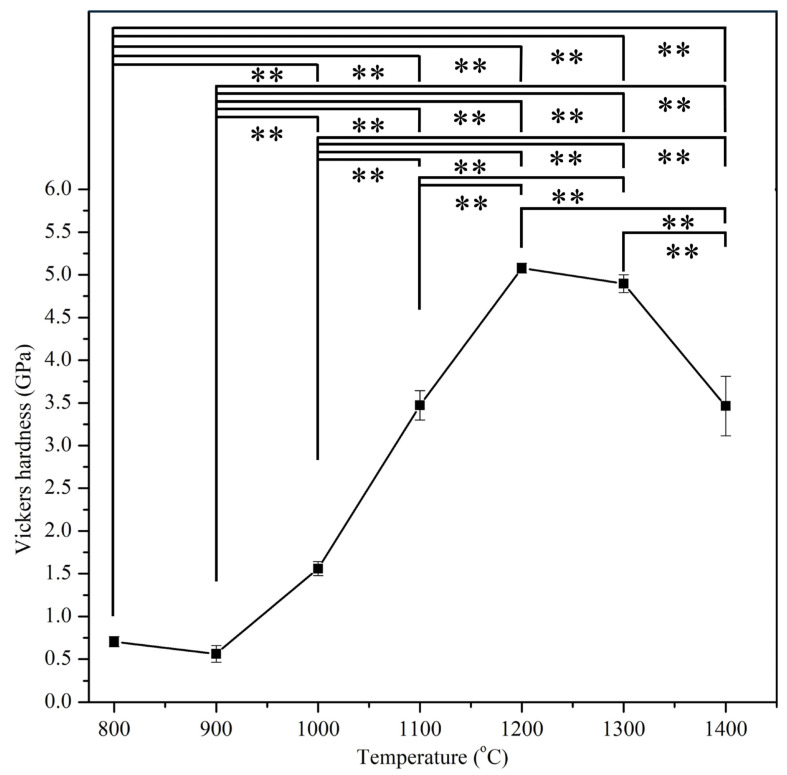
Vickers hardness of E-HA blocks sintered at different temperatures. ** indicates *p* < 0.01.

Figure 5 presents the Vickers hardnesses of E-HA compacts sintered at temperatures ranging from 800 to 1400 °C, revealing hardness values in the range of 0.56 to 5.08 GPa. The highest hardness was observed at 1200 °C (5.08 GPa), exceeding that of human tooth enamel (2.61–3.13 GPa) [30]. Beyond 1200 °C, hardness values decreased with increasing sintering temperature.

**Figure 6:**
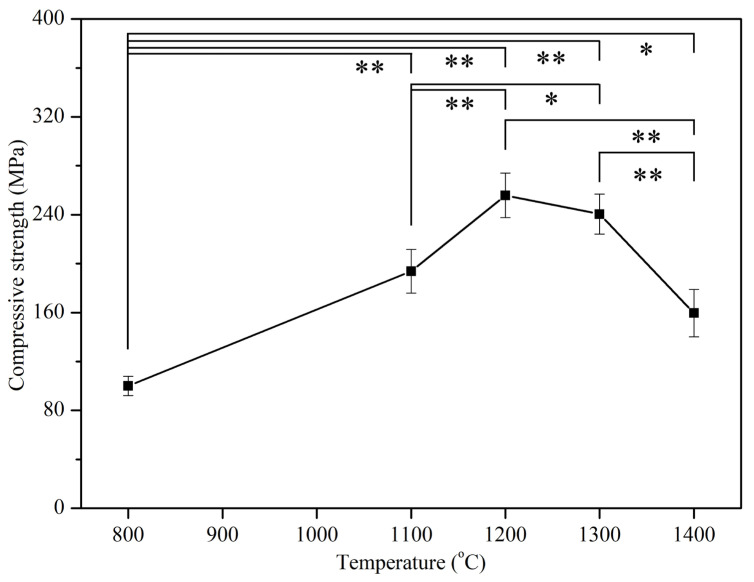
Compressive strengths of E-HA blocks sintered at different temperatures. * indicates *p* < 0.05, and ** indicates *p* < 0.01.

Figure 6 illustrates the relationship between the compressive strength of E-HA compacts and sintering temperatures, mirroring the trend observed in hardness. Below 1200 °C, compressive strength increased with higher temperatures, peaking at 256 MPa at 1200 °C, notably higher than natural bone (170–193 MPa) [31]. However, temperatures above 1200 °C saw a decline in compressive strength with increasing temperature.

**Figure 7:**
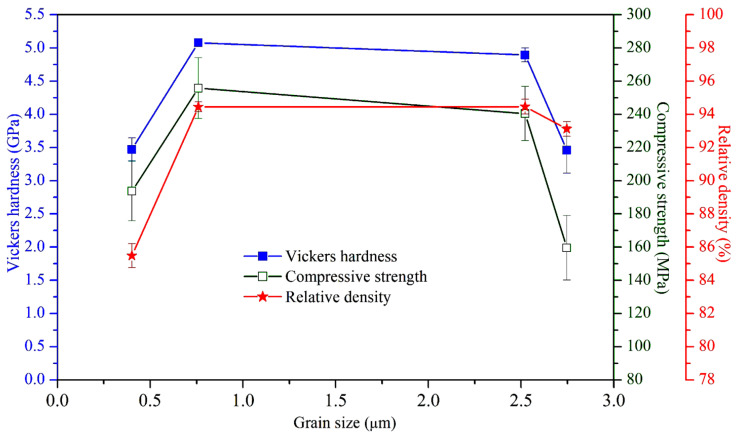
Variation in Vickers hardness, compressive strength, and relative density of sintered E-HA compacts as a function of grain size.

Figure 7 depicts the relationship among relative density, Vickers hardness, compressive strength, and grain size of sintered E-HA compacts. At a grain size of 0.760 μm (at 1200 °C), the maximum hardness (5.08 GPa) and compressive strength (255.79 MPa) were observed. Grain sizes smaller than 0.760 μm predominantly affected hardness and compressive strength through relative density, showing decreases when relative density was lower. As grain size increased from 0.760 μm to 2.522 μm (at 1300 °C), hardness and compressive strength decreased due to grain coarsening, despite a slight increase in relative density. The further increase in grain size to 2.746 μm (at 1400 °C) significantly reduced both hardness and compressive strength.

**Figure 8:**
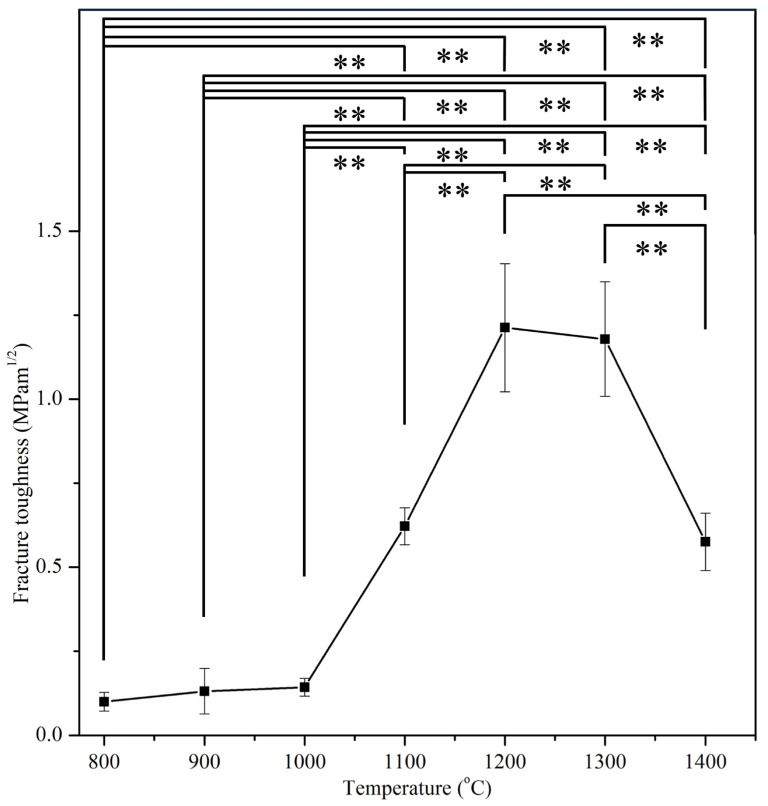
Fracture toughness of E-HA blocks sintered at different temperatures. ** indicates *p* < 0.01.

Figure 8 presents the fracture toughness of E-HA samples at various sintering temperatures, showing a trend similar to their hardness and compressive strengths. At 1200 °C, fracture toughness peaked at 1.21 MPa·m^0.5^, declining at temperatures above 1200 °C due to increased grain size. Lower sintering temperatures (800–1000 °C) exhibited relatively lower fracture toughness (0.10–0.14 MPa·m^0.5^) (*p* < 0.01), attributed to the presence of numerous pores in the samples.

### 3.5. Antibacterial Activity Test

**Figure 9:**
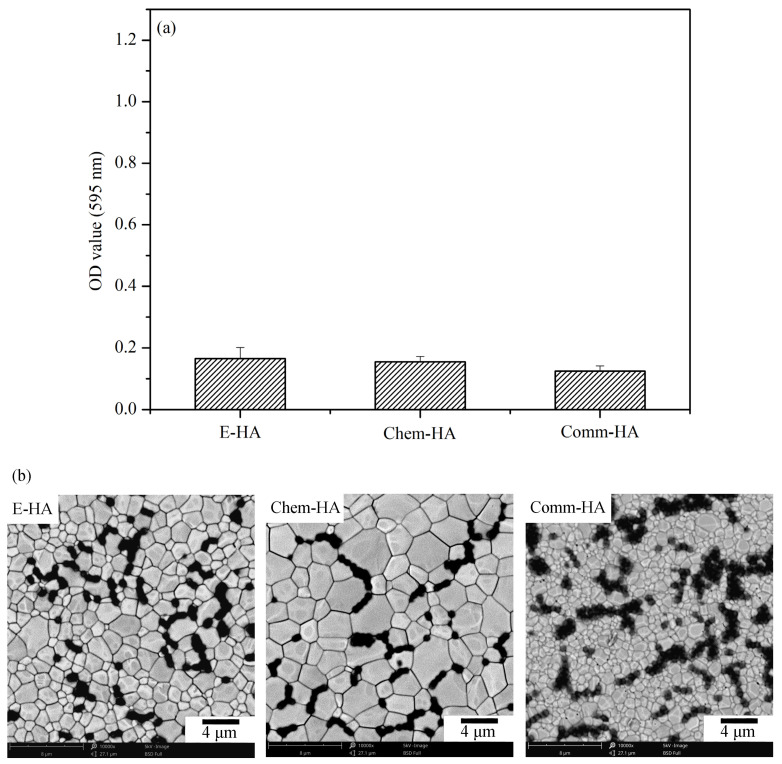
OD values of crystal violet absorbance (**a**) and SEM images (**b**) on the surfaces of E-HA, Chem-HA, and Comm-HA samples after culturing *Streptococcus mutans* for 20 h.

*Streptococcus mutans* was selected for this study because it is a primary pathogen responsible for dental caries [32,33], making it highly relevant for evaluating antibacterial efficacy in materials intended for oral and dental applications. Given its prominence in carious lesions and its well-documented role in oral health, using this bacterium provides a focused and meaningful assessment of the antibacterial properties of the HA samples. The E-HA sample sintered at 1200 °C, exhibiting superior mechanical properties, was evaluated for antibacterial activity against *Streptococcus mutans*. Additionally, Chem-HA (prepared from chemical reagent-grade calcium carbonate) and Comm-HA (commercial HA) samples sintered at 1200 °C were used as control groups. The surfaces of E-HA, Chem-HA, and Comm-HA specimens were cultured with *Streptococcus mutans* for 20 h, and their antibacterial efficacy was assessed through crystal violet absorption (Figure 9a) and SEM images (Figure 9b). The results indicated similar optical density (OD) values among all samples (Figure 9a), suggesting comparable antibacterial activity (*p* > 0.05) against *Streptococcus mutans*. The OD values measured from the bacterial cultures were consistently low, with all values falling below 0.2. This indicates that all types of HA samples exhibited significant antibacterial efficacy, effectively inhibiting bacterial growth. The comparable bacterial counts and low OD values suggest that the source of HA—whether derived from eggshells, reagent-grade calcium carbonate, or commercial sources—does not significantly affect the antibacterial properties. These findings highlight the potential of eggshell-derived HA as a viable and effective material for biomedical applications, particularly in preventing bacterial infections. SEM images (Figure 9b) further confirmed similar bacterial densities on the surfaces of all three HA samples, indicating effective antibacterial properties against *Streptococcus mutans*.

## 4. Discussion

The XRD analysis confirmed that the initial E-HA green compact was a single-phase HA with low crystallinity, as expected for HA synthesized via wet precipitation methods [19,34]. The observed phase transformation to β-TCP with increasing sintering temperatures aligns with the literature reports indicating such transitions at elevated temperatures. The additional presence of α-TCP at 1400 °C corroborates previous findings of phase changes at high sintering temperatures. The increase in crystallinity with temperature, reaching up to 95% at temperatures above 1000 °C, is consistent with the sharpening of diffraction peaks due to enhanced atomic order.

The relative density results show a clear trend of increased densification with higher sintering temperatures, peaking at 1200 °C. The observed density drop at 1400 °C is attributed to the formation of α-TCP, which has a lower density compared to HA. This is consistent with Kamalanathan et al. [7], who reported similar density reductions due to the presence of secondary phases. The consolidation and densification process observed in this study is typical for HA sintered at these temperatures, as also noted by Thuault et al. [35].

FE-SEM analysis revealed the progression of densification and grain growth with increasing sintering temperatures. The initial nano-sized rod-shaped particles transitioned to well-defined grains with clear boundaries at higher temperatures, corroborating the increase in relative density. The significant grain growth observed at 1300 °C and 1400 °C aligns with the substantial coarsening typically observed in sintered ceramics at elevated temperatures. The presence of trace elements in E-HA, potentially inhibiting grain growth, is an interesting finding that warrants further investigation, as suggested by previous studies. Ramesh et al. [6] reported that HA samples synthesized via precipitation and sintered between 1000 and 1400 °C exhibited grain sizes ranging from 2 μm to 16 μm, significantly larger than those observed in the current study. The presence of trace elements (Na, Mg, Sr, etc.) in the E-HA synthesized from eggshells in this study may act as barriers to atomic diffusion, potentially inhibiting grain growth. Kamalanathan et al. [7] also suggested that HA synthesized from eggshells could inhibit grain growth.

The mechanical properties analysis showed that the highest hardness and compressive strength were achieved at 1200 °C, with values comparable to or exceeding those of natural tooth enamel and bone. The decline in these properties at higher temperatures is attributed to grain coarsening and the formation of secondary phases. These findings align with Kamalanathan et al. [7], who noted that hardness is primarily affected by grain growth when grain sizes exceed 1.72 μm. Additionally, the decrease in hardness and compressive strength at higher temperatures may be attributed to the formation of secondary phases (β-TCP and α-TCP) during HA decomposition [36,37]. Ou et al. [38] found that after sintering HA at 1400 °C for 1 h, part of the HA phase transformed into α-TCP. Through TEM observation, it was discovered that the α-TCP structure contained numerous nanopore defects, leading to a decrease in strength and hardness. In this study, when the sintering temperature of E-HA was increased from 1300 °C to 1400 °C, the microhardness and compressive strength significantly decreased, which may also be attributed to the nanopore defects in the α-TCP structure.

The fracture toughness values of 1.21 MPa·m^0.5^ and 1.18 MPa·m^0.5^ at 1200 °C and 1300 °C, respectively, surpassed that of human tooth enamel (0.94 MPa·m^0.5^) [35,39,40]. Notably, the fracture toughness values obtained in this study surpass those (0.35–1.15 MPa·m^0.5^) reported by Kamalanathan et al. [7], indicating the superior mechanical properties of the synthesized E-HA.

The antibacterial activity tests against *Streptococcus mutans* demonstrated that all HA samples, regardless of their source, exhibited significant antibacterial efficacy. The low OD values and similar bacterial densities observed across E-HA, Chem-HA, and Comm-HA samples suggest that the antibacterial properties are not significantly affected by the source of HA. This aligns with previous findings that the release of calcium and phosphate ions plays a crucial role in the antibacterial effect [41]. The results highlight the potential of E-HA as an effective material for biomedical applications, particularly in preventing bacterial infections in dental and oral health contexts.

While our study provides valuable insights into the mechanical properties and antimicrobial activity of HA sintered at various temperatures, several methodological limitations should be considered. Firstly, although we used standardized testing protocols and ensured consistency by employing the same operator for all experiments, variations in sample preparation and handling could introduce minor discrepancies. Secondly, while we conducted comprehensive statistical analyses to assess differences among sintering temperatures and antibacterial effects, other factors such as variations in sample size or experimental conditions might influence the outcomes. Additionally, the study focused on a specific bacterial strain (*Streptococcus mutans*) for antimicrobial evaluation, which may not fully represent broader microbial interactions relevant to clinical applications. Finally, the study did not explore long-term stability or degradation characteristics of the HA samples, which are critical for assessing their suitability in biomedical contexts over extended periods. Addressing these limitations in future research could further refine the understanding and application of HA-based materials in biomedical settings.

## 5. Conclusions

In this study, hydroxyapatite (HA) powders synthesized from eggshells demonstrated varying microstructural and mechanical properties across different sintering temperatures (800–1400 °C). The increase in temperature resulted in phase transformations, with β-TCP appearing between 800 and 1300 °C and α-TCP additionally observed at 1400 °C alongside HA and β-TCP. Higher sintering temperatures contributed to increased relative density, reaching a peak of 94.5% at 1200 and 1300 °C, although a slight decline was observed at 1400 °C due to α-TCP presence. Grain size also escalated from 0.024 μm at 800 °C to 2.746 μm at 1400 °C. Notably, HA sintered at 1200 °C exhibited superior mechanical properties, including the highest hardness (5.08 GPa), compressive strength (255.79 MPa), and fracture toughness (1.21 MPa·m^0.5^). Our findings underscore that mechanical performance was predominantly influenced by relative density at temperatures below 1200 °C, while above this threshold, grain size and phase transformations played significant roles. These insights advance our understanding of optimizing HA production for biomedical applications, emphasizing the importance of precise sintering temperature control to tailor material properties effectively.

## Figures and Tables

**Table 1:** Summary of experimental groups, sample numbers, and tests performed.

Group	Sintering Temperature (°C)	Sample Number	Test Performed
E-HA	800 °C, 900 °C, 1000 °C, 1100 °C, 1200 °C, 1300 °C, and 1400 °C	Five specimens for each sintering temperature	Relative density
E-HA	1100 °C, 1200 °C, 1300 °C, and 1400 °C	Five specimens for each sintering temperature	Grain size
E-HA	800 °C, 900 °C, 1000 °C, 1100 °C, 1200 °C, 1300 °C, and 1400 °C	Five specimens for each sintering temperature	Vickers hardness
E-HA	800 °C, 1100 °C, 1200 °C, 1300 °C, and 1400 °C	Five specimens for each sintering temperature	Compressive strength
E-HA	800 °C, 900 °C, 1000 °C, 1100 °C, 1200 °C, 1300 °C, and 1400 °C	Five specimens for each sintering temperature	Fracture toughness
E-HA	1200 °C	Three specimens	Antimicrobial activity
Chem-HA	1200 °C	Three specimens	Antimicrobial activity
Comm-HA	1200 °C	Three specimens	Antimicrobial activity

## Data Availability

The original contributions presented in the study are included in the article, further inquiries can be directed to the corresponding author.
